# Genetic contribution to severe COVID-19 in adults under 60 years without major comorbidities in the German National Pandemic Cohort Network (NAPKON)

**DOI:** 10.1186/s40246-025-00904-9

**Published:** 2026-01-23

**Authors:** Ayda Abolhassani, T. Madhusankha Alawathurage, Axel Schmidt, Fabian Brand, Laura L. Kilarski, Heidi Altmann, Edgar Dahl, Sandra Frank, Siri Göpel, Frank Hanses, Johannes Christian Hellmuth, Christian Herr, Achim J. Kaasch, Robin Kobbe, Margarethe Justine Konik, Isabell Pink, Christoph Römmele, Jan Rupp, Christian S. Scheer, Marc A. Schneider, Christoph Stellbrink, Hans Christian Stubbe, Phil-Robin Tepasse, Andreas Teufel, István Vadász, Maria J. G. T. Vehreschild, Martin Witzenrath, Gabriele Anton, Isabel Bröhl, Susanne Herold, Thomas Illig, Steffi Jiru-Hillmann, Peter Krawitz, Lazar Mitrov, Alexandra Philipsen, Sina M. Pütz, Markus M. Noethen, Peter Nuernberg, Jens-Peter Reese, Olaf Riess, Stefan Schreiber, Joachim Schultze, Fridolin Steinbeis, J. Janne Vehreschild, Christian Wildberg, Kerstin U. Ludwig, Eva C. Schulte

**Affiliations:** 1 Department of Psychiatry and Psychotherapy, Faculty of Medicine & University Hospital Bonn, University of Bonn, Bonn, Germany; 2 Institute of Human Genetics, Faculty of Medicine & University Hospital Bonn, University of Bonn, Bonn, Germany; 3 Institute of Genomic Statistics and Bioinformatics, Faculty of Medicine, University of Bonn & University Hospital Bonn, Bonn, Germany; 4 Department of Internal Medicine I, University Hospital Carl Gustav Carus TU Dresden, Dresden, Germany; 5 Biobank Dresden, Faculty of Medicine and University Hospital Carl Gustav Carus, TUD Dresden University of Technology, Dresden, Germany; 6 Institute of Pathology, University Hospital Aachen, RWTH Aachen University, Aachen, Germany; 7 RWTH Centralized Biomaterial Bank (RWTH cBMB), Medical Faculty of RWTH Aachen University, 52074 Aachen, Germany; 8 Department of Anaesthesiology, LMU University Hospital, LMU Munich, Munich, Germany; 9 Department of Internal Medicine I, Division of Infectious Diseases, University Hospital Tübingen, Tübingen, Germany; 10 German Centre for Infection Research (DZIF) Clinical Research Unit for Healthcare Associated and Antibiotic Resistant Bacterial Infections, Tübingen, Germany; 11 Department for Infection Control and Infectious Diseases, University Hospital Regensburg, Regensburg, Germany; 12 Department of Medicine III, University Hospital, LMU Munich, Munich, Germany; 13 COVID-19 Registry of the LMU Munich (CORKUM), University Hospital, LMU Munich, Munich, Germany; 14 Department of Internal Medicine V – Pulmonology, Allergology, Intensive Care Medicine, Saarland University, Saarbrücken, Germany; 15 Institute of Medical Microbiology and Hospital Hygiene, Medical Faculty, Otto von Guericke University Magdeburg, Magdeburg, Germany; 16 Institute for Infection Research and Vaccine Development (IIRVD), Center for Internal Medicine, University Medical Center Hamburg-Eppendorf, Hamburg, Germany; 17 Department of Infectious Disease Epidemiology, Bernhard Nocht Institute for Tropical Medicine, Hamburg, Germany; 18 Department of Infectious Diseases, West German Centre of Infectious Diseases, University Medicine Essen University Hospital Essen, University Duisburg-Essen, Essen, Germany; 19 Department of Respiratory Medicine and Infectious Diseases, Hannover Medical School, Hannover, Germany; 20 German Center for Lung Research (DZL), Biomedical Research in Endstage and Obstructive Lung Disease Hanover (BREATH), Hannover, Germany; 21 Gastroenterology and Infectious Diseases, Faculty of Medicine, University Hospital of Augsburg, Augsburg, Germany; 22 Infectious Disease Clinic and Institute of Med. Microbiology, University-Hospital Schleswig-Holstein/Campus Lübeck, Lübeck, Germany; 23 Department of Anesthesiology, Intensive Care Medicine, Emergency Medicine and Pain Medicine, University Medicine Greifswald, Greifswald, Germany; 24 Translational Research Unit, Thoraxklinik at Heidelberg University Hospital, Heidelberg, Germany; 25 Translational Lung Research Center Heidelberg (TLRC), German Center of Lung Research (DZL), Heidelberg, Germany; 26 Academic Department of Cardiology and Internal Intensive Care Medicine, Bielefeld University, Medical School and University Medical Center East Westphalia-Lippe, Klinikum Bielefeld, Bielefeld, Germany; 27 Department of Medicine II, University Hospital, LMU Munich, Munich, Germany; 28 Department of Medicine B for Gastroenterology, Hepatology, Endocrinology and Clinical Infectiology, University Hospital Muenster, Münster, Germany; 29 Division of Hepatology, Department of Medicine II, Medical Faculty Mannheim, Heidelberg University, Mannheim, Germany; 30 Department of Internal Medicine, Justus Liebig University, Universities of Giessen and Marburg Lung Center (UGMLC), German Center for Lung Research (DZL), Giessen, Germany; 31 Excellence Cluster Cardio-Pulmonary Institute (CPI), Giessen, Germany; 32 Institute for Lung Health (ILH), Justus Liebig University Giessen, Giessen, Germany; 33 Department of Internal Medicine, Infectious Diseases, University Hospital Frankfurt, Goethe University Frankfurt, Frankfurt am Main, Germany; 34 Department I for Internal Medicine, Faculty of Medicine and University Hospital of Cologne, University of Cologne, Cologne, Germany; 35 German Center for Infection Research (DZIF), Partner-Site Cologne-Bonn, Cologne, Germany; 36 Department of Infectious Diseases, Respiratory Medicine and Critical Care, Charité-Universitätsmedizin Berlin, Corporate Member of Freie Universität Berlin and Humboldt-Universität zu Berlin, Berlin, Germany; 37 Berlin, Germany; 38 Berlin, Germany; 39 Medical School OWL, Bielefeld University, Bielefeld, Germany; 40 Faculty of Medicine and University Hospital Cologne, Department I of Internal Medicine, Division of Infectious Diseases, University of Cologne, Cologne, Germany; 41 Department of Medicine V, Internal Medicine, Infectious Diseases and Infection Control, Universities of Giessen and Marburg Lung Center (UGMLC), German Center for Lung Research (DZL), German Center for Infection Research (DZIF), Justus-Liebig University Giessen (JLU), Giessen, Germany; 42 Hannover Unified Biobank, Hannover Medical School, Hannover, Germany; 43 Institute for Clinical Epidemiology and Biometry, University of Würzburg, Würzburg, Germany; 44 Cologne Center for Genomics (CCG), University of Cologne, Cologne, Germany; 45 Institute for Medical Data Science, University Hospital Würzburg, Würzburg, Germany; 46 DFG NGS Competence Center Tübingen (NCCT), University of Tübingen, Tübingen, Germany; 47 Institute of Medical Genetics and Applied Genomics, University of Tübingen, Tübingen, Germany; 48 Internal Medicine Department I, University Hospital Schleswig-Holstein Campus Kiel, Kiel, Germany; 49 Genomics and Immunoregulation, Life & Medical Sciences (LIMES) Institute, University of Bonn, Bonn, Germany; 50 PRECISE Platform for Genomics and Epigenomics, Deutsches Zentrum für Neurodegenerative Erkrankungen (DZNE) e.V. and University of Bonn, Bonn, Germany; 51 Systems Medicine, Deutsches Zentrum für Neurodegenerative Erkrankungen (DZNE) e.V., Bonn, Germany; 52 Department II of Internal Medicine, Hematology/Oncology, Goethe University, Frankfurt, Germany; 53 Institute of Psychiatric Phenomics & Genomics, LMU University Hospital, LMU Munich, Munich, Germany; 54 German Center for Mental Health (DZPG), Partner Site Munich-Augsburg, Munich, Germany; 55 Institute of Virology, Technical University of Munich/Helmholtz Munich, Munich, Germany

## Abstract

While genome-wide association studies (GWAS) have linked common genetic variants to COVID-19 susceptibility and severity, rare high-impact variants may also contribute to phenotypic heterogeneity. Inborn errors of type I interferon immunity (IFN-I-IEIs), including X-linked *TLR7* deficiency, account for ~ 2% of critical COVID-19 cases. In this study, we investigated rare potentially deleterious variants in IFN-I-IEI and GWAS-prioritized genes in young, severely affected COVID-19 patients from the German National Pandemic Cohort Network (NAPKON). Genome sequencing was performed on 110 hospitalized COVID-19 patients, including 82 males and 28 females, all under 60 years of age and without relevant pre-existing medical conditions. Rare potentially deleterious variants in *TLR7* and 25 additional IFN-I-IEI genes, as well as 23 GWAS risk genes for COVID-19 severity, were analyzed based on allele frequency, predicted functional impact, and inheritance pattern models and subsequently classified based on the American College of Medical Genetics and Genomics (ACMG) criteria. Polygenic Risk Scores (PRS) were additionally calculated as an exploratory and case-only analysis to assess the contribution of common variant-derived genetic predisposition for severe COVID-19. Consistent with prior findings from other studies in German cohorts, no candidate variants or large deletions were identified in *TLR7*. However, 7 variants of uncertain significance in IFN-I-IEI genes as well as 13 candidate variants of potential deleterious effect in GWAS risk genes were present in 19 individuals (17.3%). We observed nominally significant differences in PRS distributions, with younger individuals (< 40 years) having higher PRS (p = 0.045) compared to older individuals, and carriers of rare variants having lower PRS compared to non-carriers (p = 0.037). These patterns are consistent with an age-dependent contribution of polygenic risk to severe COVID-19 and a potentially lower polygenic burden among rare-variant carriers, although confirmation in larger well-controlled cohorts will be required. The candidate variants identified in IFN-I-IEI and GWAS risk genes represent targets for further functional studies to clarify their potential contribution to disease risk. These findings highlight the need for future integrative genomic approaches to better understand the joint contribution of common and rare variants to COVID-19 severity.

**Supplementary Information:**

The online version contains supplementary material available at 10.1186/s40246-025-00904-9.

## Introduction

Severe acute respiratory syndrome coronavirus 2 (SARS-CoV-2) infection represents one of the greatest recorded challenges to global healthcare to date, with hundreds of millions of cases and at least 7 million associated deaths worldwide [1]. SARS-CoV-2 causes coronavirus disease 2019 (COVID-19) with highly heterogeneous clinical manifestations, ranging from asymptomatic infection to severe respiratory failure [2], and the reason underlying these inter-individual differences is yet to be completely understood. Although established demographic and clinical factors (e.g., advanced age [3]**,** male sex [4, 5], obesity, existing medical conditions [6, 7], or auto-antibodies [8]) correlate with COVID-19 severity, these risk factors do not fully explain the variability in disease outcomes. Increasing evidence suggests that host genetics play an important role in shaping infection susceptibility and disease severity [9], enabling insights into COVID-19 pathogenesis and informing therapeutic approaches.

As with many common disorders, COVID-19 is genetically complex, involving variants across the entire allelic spectrum. Genome-wide association studies (GWAS) have identified common variants in more than 70 loci associated with COVID-19 severity and susceptibility, typically characterized by an allele frequency (AF) greater than 1% and low effect sizes (odds ratio = 0.5–2.4) [10–16]. These loci include potentially causal genes involved in the type I interferon (IFN) pathway, such as *IFNAR2, OAS1, TYK2, JAK1, IRF1,* and IFNα-coding genes [16]. On the other side of the allelic spectrum, exome sequencing studies in young patients with severe COVID-19 have also uncovered rare predicted loss-of-function variants (pLOF) in genes associated with inborn errors of type I IFN immunity (IFN-I-IEIs) [17, 18]**,** further highlighting the role of this antiviral pathway in disease pathogenesis. The best-established risk gene for severe COVID-19, first identified in individual pedigrees and subsequently replicated through rare variant association approaches, is the X-chromosomal toll-like receptor 7 gene (*TLR7*) [19–21]. *TLR7* is an important part of innate viral immunity, encoding an endosomal receptor that recognizes single-stranded RNA viruses, leading to upregulation of the type 1 and type 2 interferon pathways [22]. Recent estimates suggest the presence of TLR7 deficiency in around 1–2% of male individuals with life-threatening COVID-19 under 60 years of age [20, 23].

Moreover, multiple lines of evidence indicate that autoimmunity to type I IFNs also contributes to critical COVID-19 pneumonia, as shown by the presence of pre-existing neutralizing autoantibodies (autoAbs) against type I IFNs in ~ 15% of critical cases, with higher prevalence in individuals over 70 years of age [8, 24, 25]. Present at low-levels (~ 0.3–1%) until a sharp increase post 70 years of age (up to 4–7% in individuals aged 80–85) [25], IFN-I autoAbs can also be found in children and young adults, where their presence is likely to reflect a germline genetic etiology, as observed in rare IEIs including *AIRE*-related autoimmune polyendocrinopathy syndrome type 1 (APS-1), *FOXP3*-related immune dysregulation (IPEX), and *RAG1/RAG2*-associated combined immunodeficiencies [26, 27]. Notably, APS-1 patients have been reported to be at high risk of severe COVID-19 [28–31]. Collectively, these findings indicate the central role of type I IFNs in protective immunity against SARS-CoV-2 and suggest that IFN-I-IEIs, including those underlying the production of autoantibodies, may account for a subset of severe COVID-19 cases in young adults.

The implication of the IFN-I pathway by both rare and common variant studies demonstrates how genetic variants with different effect sizes can converge on the same biological pathway contributing to COVID-19 severity. This raises the possibility that other genes prioritized by GWAS may also harbor rare high-impact variants that contribute to severe disease in a monogenic manner. Building on this hypothesis, and on prior epidemiological evidence that severe COVID-19 in young individuals without comorbidities may represent patients with higher genetic risk or monogenic predisposition to severe outcome [32], we aimed to identify potentially deleterious variants with large effect sizes in (i) known IEI genes that affect both production or response to type I IFNs, and (ii) GWAS-prioritized genes for severe COVID-19, within a cohort of young hospitalized patients without relevant pre-existing medical conditions from Germany.

## Methods

### Patient selection

The National Pandemic Cohort Network (NAPKON) was established in early 2020 as part of the German Network of University Medicine to develop the most comprehensive COVID-19 cohort in Germany. It includes over 7000 participants across all healthcare sectors, with participants enrolled in three complementary cohort platforms (cross-sectoral (SUEP), high-resolution (HAP), and population-based (POP)) where they were monitored from the initial infection for up to three years. Comprehensive phenotypic data were collected, including detailed clinical and imaging data as well as quality-of-life assessments and other patient-reported outcomes [33]. To investigate rare variants predisposing young individuals to severe COVID-19 in this study, we obtained biomaterials (DNA samples or buffy coats) and phenotypic data from NAPKON participants who met the following inclusion criteria (i) age under 60 years; (ii) absence of significant pre-existing medical condition (including cardiovascular, lung, kidney, liver, neurologic/psychiatric diseases; type 1 diabetes; active cancer; organ transplant; rheumatologic/immunologic disorders, HIV infection), and (iii) a minimum WHO severity score [34] of 4 within the first eight weeks of infection. A total of 110 individuals across all three platforms from 22 university hospitals across Germany, fulfilled these criteria and were included in the analysis. Cohort demographics are presented in Table 1 and Supplementary Table 1.

**Table 1:** Patient demographics

Features	Count (N = 110)	% of total
*Sex*		
Male	82	74.5
Female	28	25.5
*Age group (years)*		
18–29	17	15.5
30–39	25	22.7
40–49	38	34.5
50–59	30	27.3
*BMI category*		
Healthy weight (18.5–24.9 kg/m^2^)	27	24.5
Overweight (25–29.9 kg/m^2^)	43	39.1
Obese (30–39.9 kg/m^2^)	23	20.9
NA	17	15.5
Smoking status*		
No	95	86.4
Yes	5	4.5
NA	10	9.1
Vaccination status**		
No	91	82.7
Yes	10	9.1
NA	9	8.2
WHO category***		
Dead (score 10)	1	0.9
Hospitalized: severe disease (scores 6–9)	23	20.9
Hospitalized: moderate disease (scores 4–5)	86	78.2
*Comorbidities*		
Cardiovascular disease	0	0
Lung disease	0	0
Diabetes (type 2)	3	2.7
Kidney disease	0	0
Liver disease	0	0
Neurologic/psychiatric disease	0	0
Cancer	0	0
Organ transplant	0	0
Rheumatologic/immunologic disease	0	0
HIV infection	0	0

*Active smoking at the time of COVID-19 diagnosis. **Refers to COVID-19 vaccination before hospitalization. ***WHO clinical progression scale for COVID-19 [32]: Hospitalized moderate disease (WHO scores 4–5): hospitalized and no oxygen therapy or oxygen therapy by mask or nasal prongs.; Hospitalized severe disease (WHO scores 6–9): hospitalized and oxygen therapy by noninvasive ventilation, intubation, or mechanical ventilation. *NA* data not available

### Ethics statement

Written informed consent was obtained from each participant. NAPKON’s study protocols and ethical guidelines have been approved by the institutional review boards of all participating study sites [33]. The specific investigations herein were approved by the ethics committee of the Medical Faculty of the University of Bonn (171/20; amended on April 11th, 2022).

### Genome sequencing (GS)

As described previously [31], library preparation and sequencing were carried out using standardized workflows at the Bonn site of the West German Genome Center (WGGC). Enrichment followed the TruSeq DNA PCR-Free protocol, with DNA fragmented to 350 bp. The resulting libraries were sequenced as 150 bp paired-end reads on an Illumina NovaSeq6000, yielding ~ 120 Gb of data per sample. Demultiplexing and FastQ file generation were conducted using bcl2fastq2 (version 2.20.0.422), and quality control (QC) metrics were assessed with FastQC (v0.11.9) and MultiQC (v1.17). The sequencing reads were then aligned to the human reference genome (GRCh38), followed by duplicate removal and variant calling for single-nucleotide variants (SNVs) and short insertion-deletions using the Illumina DRAGEN platform (software versions 3.5.7 and 3.6.3). The generated gVCFs were then used to perform joint variant calling across all samples using a slightly modified version of GLnexus (v1.3.1) with the "gatk" setting, to create a raw cohort VCF. Modifications to the standard GLnexus pipeline incorporated community-driven enhancements to optimize variant calling in haploid regions, which are handled differently by GATK and DRAGEN.

### Sample QC and ancestry PCA

Sample QC and ancestry principal component analysis (PCA) were performed with PLINK v1.9 if not stated otherwise [35]. Sex concordance was assessed using the –check-sex flag, with inbreeding coefficient (F) thresholds of > 0.8 for males and < 0.2 for females. Samples falling out of the broader range of − 0.2 to 0.2 were identified as potential mismatches. Sample contamination was checked using VerifyBamID [36], and kinship analysis was performed using KING [37], with kinship coefficients ≥ 0.044 considered as relatedness. All samples had genome-wide coverage of ≥ 97% at 10X, with a mean depth of > 30X. Read alignment rates were > 99%, with < 5% MAPQ = 0 reads. No samples were excluded for sex discrepancies, contamination, or relatedness and all the samples had a call rate >  = 98%. For population structure and ancestry assessment, genotype data were converted from VCF to PLINK binary format, preserving allele ordering and processing sex chromosome regions with –split-x. SNV pruning was conducted to limit the variants to those outside of regions with high linkage disequilibrium (LD) using a sliding window of 50 SNVs, step size 5, and variant inflation factor (VIF) threshold of 1.5 (LD r^2^ ≈ 0.33). Variants with AF < 0.1 were excluded prior to PCA. For ancestry inference, samples were combined with 1000 Genomes Project (1000G) reference panel data [38], where only overlapping sets of high-quality variants were considered and pruned. PCA was conducted using PLINK's –pca option, and individuals were annotated by cohort (study vs. 1000G). The first 20 PCs were then used as features to train a random forest (RF) classifier in order to identify samples with “known” population labels and 1000G super-population labels as outcomes as described in the GnomAD ancestry inference documentation. For polygenic risk score (PRS) calculation, European ancestry individuals were defined based on the labels assigned by the RF classifier as the highest-vote class (EUR cluster in Supplementary Fig. 3). A total of 85 unrelated individuals of inferred European ancestry were identified.

### Polygenic risk score calculation

PRS-CS (version 1.0.0; default setting) [39] was applied to the summary statistics of European-ancestry individuals from the largest currently available GS-based GWAS for COVID-19 severity [14] (no known sample overlap with the NAPKON cohort), using the UK Biobank LD reference panel as described previously [31]. The resulting PRS predictor included 967,463 variants. PRS was then calculated using the ‘–score’ function in PLINK (version 1.9) for variants with call rate > 98%.

### Association analyses

Associations between PRS and age group (< 40 vs ≥ 40 years), and rare variant carrier status, were tested using logistic regression models with age group or carrier status as the dependent variable and PRS as the primary predictor, adjusting for sex and ten ancestry principal components. Analyses were performed in both the full cohort (n = 110) and the PCA-defined European ancestry subset (n = 85). BMI and smoking were added as covariates in secondary models restricted to individuals for whom this information was available (BMI: n = 94; BMI + smoking: n = 89) (Supplementary Table 6). P-values were calculated using Wald tests.

### Variant annotation and filtering

Ensembl BioMart was used to generate a BED file with the start and end genomic coordinates of the candidate genes (hg38), extended by 200 bp upstream and downstream of their 5'/3’ untranslated regions (UTR). Using bcftools, the QCed cohort VCF file from the 110 individuals was subset to retain only variants within the candidate gene regions and further filtered to exclude variants observed with AF ≥ 10% in the cohort. The resulting filtered VCF file was used for annotation and subsequent rare variant analysis.

Functional annotation of variants was performed using the command-line version of Ensembl Variant Effect Predictor [40] (VEP version 113) with external in silico predictions integrated as plugins (CADD (v1.7) [41], REVEL [42], AlphaMissense [43], LOFTEE [44], and SpliceAI [45]. The "pick_allele_gene" option was applied to report a single, most biologically relevant consequence per gene for each variant allele. Allelic balance thresholds were set at > 95% for homozygous or hemizygous variants and at 25–75% for heterozygous variants, with a minimum read count of 4 required for both reference and alternative alleles.

**Fig. 1:**
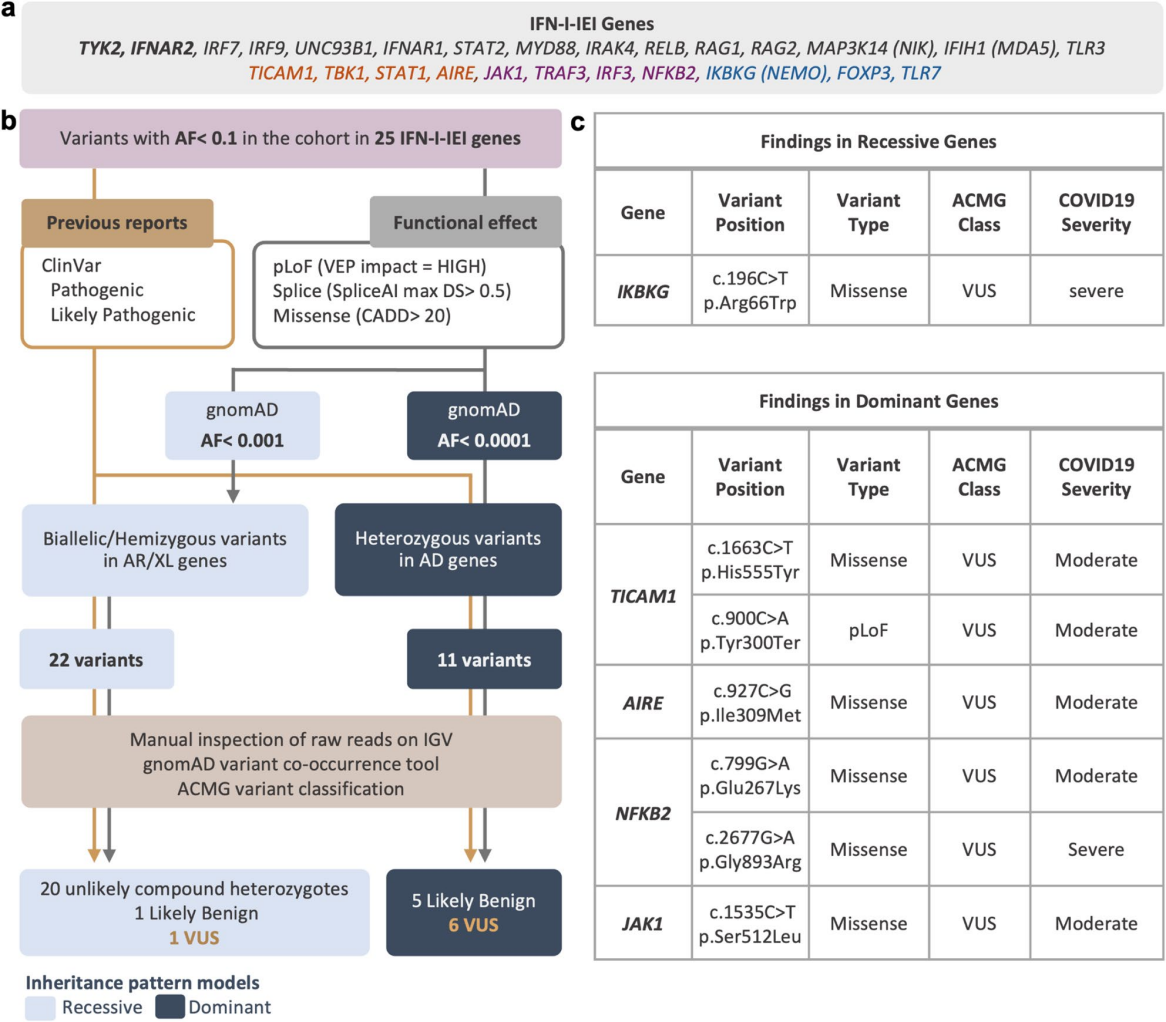
Variant filtering strategy and findings in the clinical approach. **a** Inborn errors of type 1 IFN immunity (IFN-I-IEI) genes included in the analysis. Font colors indicate inheritance: AR (gray), AD/AR (orange), AD (purple), and X-linked (blue). Overlap with the GWAS genes is shown in bold. Alternative (non-HGNC) gene symbols are indicated in parentheses. **b** The filtering process to identify clinically relevant variants in 25 IFN-I-IEI genes from genome sequencing data. Variants were selected based on pathogenic entries in ClinVar andpredicted functional effects, and then filtered according to allele frequency thresholds specific to recessive and dominant genes. This was followed by manual review using IGV and the gnomAD variant co-occurrence tool to assess variant quality and cis/trans phase before final classification according to ACMG guidelines. **c** Characteristics of variants of uncertain significance (VUS) identified in genes with reported dominant or recessive patterns of inheritance in the cohort. *AF* allele frequency, *pLoF* potential loss of function variant, *VEP* Variant Effect Predictor, *VUS* variant of uncertain significance, *IGV* Integrative Genomics Viewer

**Fig. 2:**
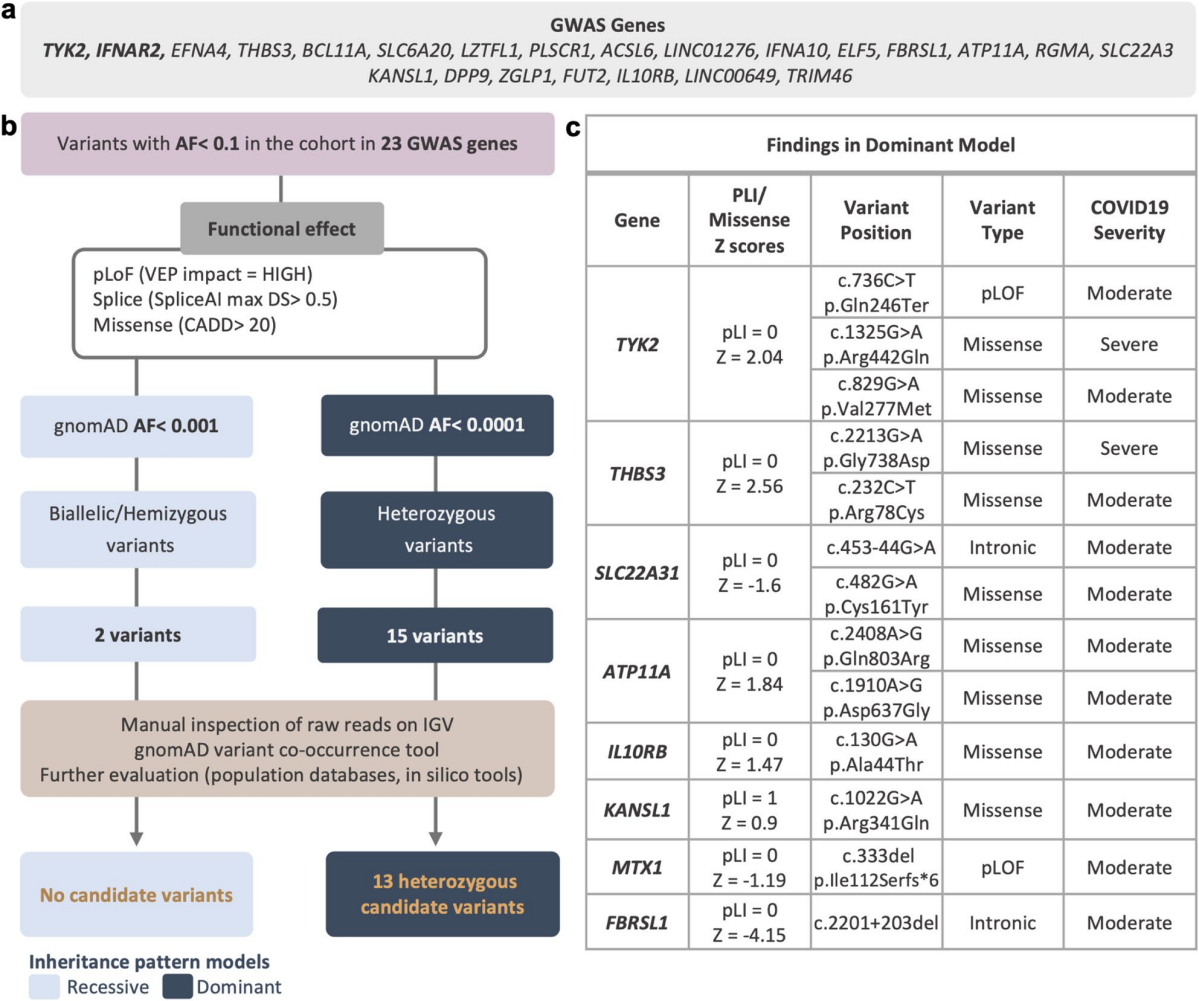
Variant filtering strategy and findings in the research approach. **a** 23 GWAS-prioritized genes for COVID-19 severity included in the analysis. Overlap with the IFN-I-IEI genes is shown in bold. **b** The filtering process is similar to the clinical approach shown in Fig. 1; except that ClinVar information and ACMG criteria were not applied, and all genes were analyzed under both recessive and dominant models. **c** The characteristics of identified variants and gene constraint information based on gnomAD. *pLoF* potential loss of function variant, *pLI* probability of loss of function intolerance based on gnomAD, *VEP* Ensemble variant effect predictor, *IGV* Integrative genomics viewer

We applied two different strategies to identify rare high-impact variants potentially relevant under either recessive or dominant patterns of inheritance, as shown in Figs. 1 and 2. The primary difference between the two inheritance modelsis the AF cutoff applied to filter variants and the zygosity of the considered variants for each model (putative biallelic/hemizygous variants in the recessive model, and heterozygous variants in the dominant model). In both models, we included predicted loss-of-function (pLoF) variants classified as “high impact” by VEP, missense variants with CADD scores > 20, and variants with SpliceAI max delta scores > 0.5. Among these, variants with gnomAD AF < 0.001 were retained for the recessive model, while only those with gnomAD AF < 0.0001 were retained for the dominant model. We then added pathogenic or likely pathogenic variants as reported by ClinVar (regardless of allele frequency) into the final variant sets for both models. In the final recessive variant set, only homozygous or hemizygous variants, as well as cases in which an individual carried more than two variants in the same gene (potential compound heterozygotes), were retained. For the dominant model, only heterozygous variants were included. Variant co-occurrence (gnomAD version 2 [46]) and/or manual inspection of raw reads in Integrative Genomics Viewer (IGV) [47] were used to determine the likelihood of variants being located on the same strand (in *cis*) as opposed to the opposite strand (in *trans*, i.e., compound heterozygous).

The above variant filtering strategy was applied to two different gene sets, corresponding to two distinct analysis approaches:

1. *Clinical approach:* established IFN-I-IEI genes were analyzed according to their reported inheritance pattern for the relevant phenotype in the Online Mendelian Inheritance in Man (OMIM) database (Supplementary Table 2). Variants identified through this approach were classified using the American College of Medical Genetics and Genomics (ACMG) criteria [48] to determine their clinical relevance (Fig. 1).
2. *Research approach:* GWAS-prioritized genes were analyzed to identify potentially deleterious variants under both recessive and dominant patterns of inheritance (Fig. 2). The same approach was also applied to the IFN-I-IEI genes to evaluate variants that might not follow the known inheritance pattern but could contribute to severe phenotype with a novel pattern of inheritance (Supplementary Table 5).

### Qualitative copy number variation (CNV) analysis at the *TLR7* locus

To screen for potential large gene-spanning deletions at the *TLR7* locus, a targeted coverage-based analysis was performed. The cohort VCF was queried for missing genotypes (GT = ./.) or absent read depth (DP = 0) across variants within chrX:12,760,551–12,980,636 in male individuals, which would be consistent with a hemizygous deletion. In addition, read-depth patterns were visually inspected in IGV to identify extended regions of markedly reduced or absent coverage which could indicate possible hemizygous deletions.

## Results

### Cohort demographics

Genome sequencing was performed on 110 individuals under the age of 60 years hospitalized due to COVID-19. Cohort characteristics are presented in Table 1 and Supplementary Table 1. The average age was 42.0 years (± 10.7 years), and 28 (25.5%) individuals were female. Based on the WHO clinical progression scale for COVID-19 [34], disease severity was moderate in 86 individuals (78%), while 23 (21%) were classified as severe cases, and 1 individual (0.91%) died from COVID-19. Consistent with existing epidemiological evidence, disease severity was higher both in older and in male individuals (Supplementary Fig. 1).

### Targeted analysis of the *TLR7* locus

As X-linked TLR7 deficiency remains the most well-established monogenic cause of severe COVID-19 to date [19, 20], we first sought to investigate the presence of potentially pathogenic variants within the *TLR7* coding sequence. No non-synonymous or potentially splice-altering variants with AF < 0.1 in the cohort were detected. We next performed a targeted coverage-based screen for large hemizygous deletions spanning *TLR7* coding region in 82 male individuals by visually inspecting sequence reads. This approach did not reveal evidence of large hemizygous deletions in the coding regions of *TLR7* (Supplementary Fig. 2).

### Clinical approach: analysis of 25 genes implicated in IFN-I-IEIs

To identify potential monogenic causes of severe COVID-19, we investigated 25 additional IFN-I-IEI genes, previously reported in patients with severe COVID-19, influenza, or other viral infections [49] (Fig. 1; Supplementary Table 2). The filtering strategy outlined in Fig. 1 was applied, and variants were classified based on ACMG criteria [48]. No pathogenic or likely pathogenic variants were identified within this gene set. However, we observed six heterozygous variants of uncertain significance (VUS) in autosomal dominant (AD) disease genes (*TICAM1 (n* = *2), NFKB2 (n* = *2), AIRE, JAK1*), as well as one hemizygous VUS in the X-linked recessive gene *IKBKG* (Fig. 1; Supplementary Table 3). None of the identified variants have been previously reported in patients with IEI or severe COVID-19 to date. Among them, two variants are of potential clinical interest. The hemizygous missense variant in *IKBKG* (c.196C > T, p.Arg66Trp) was identified in a 46-year-old male patient with severe COVID-19 (WHO score 6: high-flow oxygen therapy). This variant has a gnomAD AF of 0.002751% (allele count:13, 0 homo/hemizygotes) and is only observed in females. It has a CADD score of 25.4, but inconsistent predictions using other in silico tools (SIFT: deleterious; MutationTaster: benign; AlphaMissense: likely benign; PolyPhen-2: possibly damaging). Pathogenic missense variants in *IKBKG* have been frequently reported to cause immunodeficiency with or without ectodermal dysplasia in males [50]. Based on the currently available evidence and according to the ACMG guidelines this variant is classified as VUS (PM2_supporting, PP2). The heterozygous missense variant in *AIRE* (c.927C > G, p.Ile309Met) was detected in a 56-year-old male patient with moderate disease (WHO score 5: oxygen therapy by mask or nasal prongs). This variant is located within the PHD1 domain of the protein, where missense variants with dominant-negative effects have been reported to cause a rare nonclassical form of autoimmune polyendocrine syndrome type 1 (APS-1), characterized by later onset, milder phenotypes, and reduced penetrance [51, 52]. This variant has a gnomAD AF of 0.01073% (allele count:173, 0 homozygotes) and multiple in silico tools predict it to be deleterious (CADD: 23.9; REVEL: 0.734; AlphaMissense: likely pathogenic; SIFT: deleterious; PolyPhen-2: possibly damaging). Notably, another nucleotide change at the same amino acid position, resulting in a threonine substitution (c.926 T > C; p.I309T), has been reported in a non-classical APS-1 patient and shown to exert a dominant-negative effect by reducing AIRE-regulated gene expression when co-expressed with the wild-type [52]. However, the available evidence is currently insufficient to determine the role of this variant in disease and it is classified as VUS based on ACMG criteria (PM1, PM5, PP3, BS1).

### Research approach: analysis of 23 GWAS-prioritized genes for COVID-19 severity

To identify potentially deleterious rare variants in genes prioritized by GWAS for COVID-19 severity, we next applied the filtering strategy outlined in Fig. 2, to 23 genes reported in the largest GS-based GWAS on COVID-19 severity published to date [14] (Supplementary Table 2). Two genes (*TYK2* and *IFNAR2*) overlapbetween the IFN-I–IEI and GWAS-prioritized gene sets. Thirteen heterozygous candidate variants in *TYK2 (n* = *3), THBS3 (n* = *2), SLC22A31 (n* = *2), ATP11A (n* = *2), MTX1, FBRSL1, IL10RB,* and *KANSL1* were identified, all under the dominant inheritance model. Of all the identified variants, 2 were pLoF, 9 were missense, and 2 were intronic variants with predicted splice-altering effects (Fig. 2; Supplementary Table 4). We used gene constraint metrics from gnomAD (probability of loss-of-function intolerance (pLI) and missense Z scores) to evaluate gene tolerance to different variant types. Both genes with pLoF variants *(TYK2* and *MTX1),* as well as the two genes with potential splice-altering variants *(SLC22A31, FBRSL1),* have pLI scores of 0, consistent with tolerance to heterozygous loss-of-function. Among genes harboring missense variants, *THBS3* showed the highest degree of missense constraint, with a Z-score of 2.56, indicating moderate intolerance to missense variation. The heterozygous c.2213G > A (p.Gly738Asp) variant identified in *THBS3* was observed in a 25-year-old female patient with severe disease (WHO score 8 to 9; mechanical ventilation). This variant is rare in the general population, with a gnomAD AF of 0.0002478% (allele count:4, 0 homozygotes) and is consistently predicted to be deleterious by multiple in silico tools (CADD: 33, REVEL: 0.9, and AlphaMissense: likely pathogenic). *THBS3* encodes thrombospondin-3, a member of the thrombospondin family of adhesive glycoproteins involved in cell to cell and cell to matrix interactions [53].

Given prior evidence that in silico–predicted deleterious variants in disease genes may reveal novel inheritance patterns upon functional validation, we additionally applied our research approach to 25 IFN-I-IEI genes to analyze each under both dominant and recessive models capturing potentially damaging variants that may follow inheritance mechanisms not yet associated with these genes. This analysis identified 17 heterozygous variants consistent with a dominant model in recessive IFN-I-IEI genes, including *IFIH1 (n* = *3), IRF7 (n* = *3), UNC93B1 (n* = *3), RELB (n* = *3), TYK2 (n* = *3), MAP3K14,* and *RAG1 *(Supplementary Table 5).

### Contribution of common-variant-derived genetic risk for severe COVID-19

**Fig. 3:**
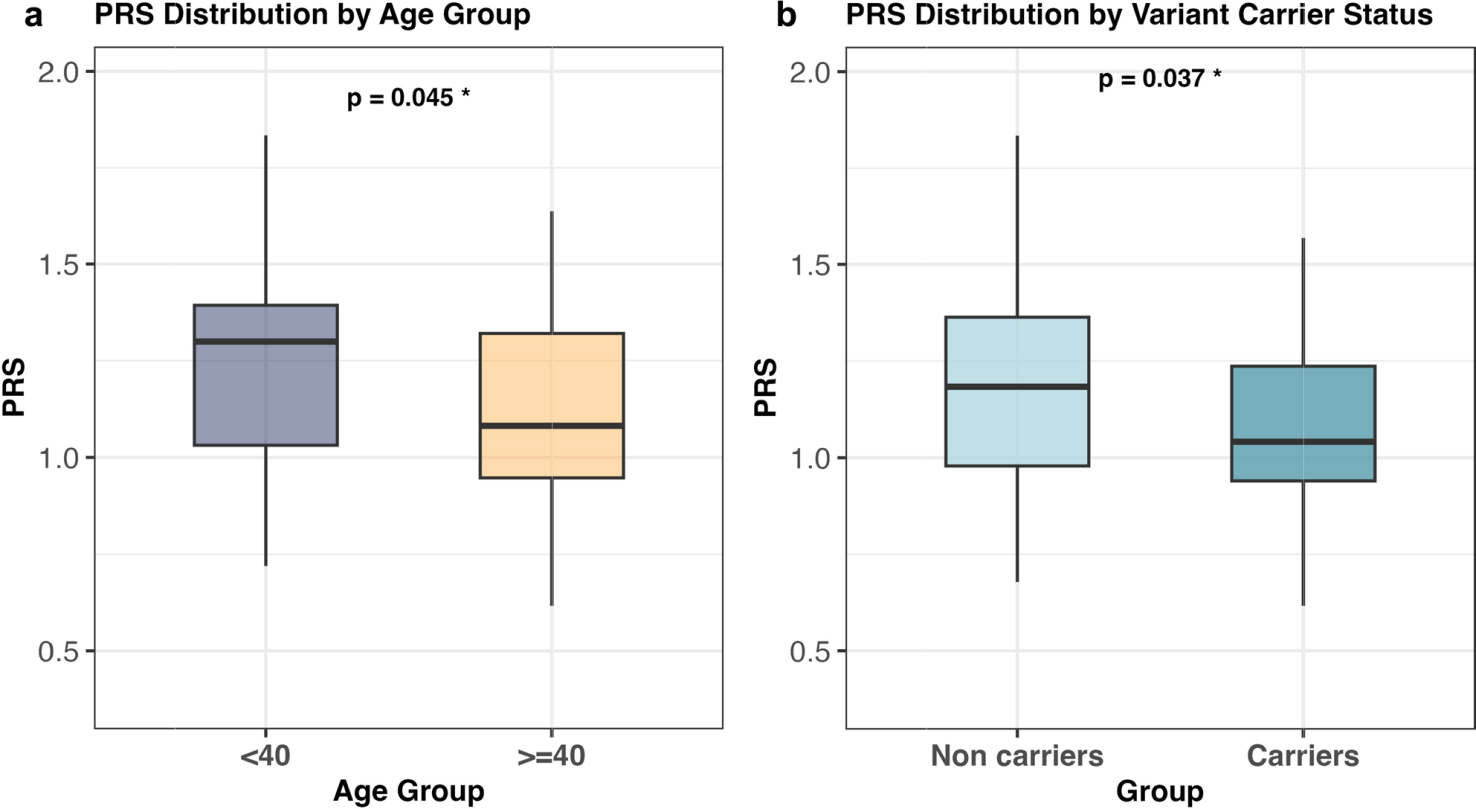
Distribution of polygenic risk scores (PRS) for severe COVID-19 by age group and variant carrier status. Box plots show the distribution of individual PRS in **a** individuals < 40 years (n = 42) versus ≥ 40 years (n = 68), and **b** carriers of at least one rare candidate variant (n = 19) versus non-carriers (n = 91). Box plot elements: box: interquartile range (IQR); dashed line: whiskers: range within 1.5 × IQR; points: outliers. *p < 0.05; Wald test

Given that age is an established independent risk factor for severe COVID-19 [3], and that additional risk factors such as comorbidities accumulate with increasing age, we hypothesized that younger individuals with severe disease may carry a higher genetic burden for COVID-19 severity, reflecting both rare and common variants with varying effect sizes. To test this, we calculated individual PRS based on the largest currently available GS-based GWAS summary statistics for COVID-19 severity [14] and examined associations in the full cohort (n = 110) and the PCA-defined European subset (n = 85) using logistic regression adjusted for sex and ten ancestry principal components. Additional sensitivity analyses were performed using fewer principal components and with additional adjustment for BMI (n = 94) and BMI plus smoking (n = 89) in individuals with available data (Supplementary Table 6). In the full cohort, higher PRS was associated with younger age (< 40 years) (OR = 0.13, 95% CI 0.02–0.96; p = 0.045), suggesting polygenic factors may contribute more substantially to COVID-19 severity in younger individuals (Fig. 3). This inverse association remained robust after adjustment for BMI (OR = 0.064; p = 0.024) and for BMI plus smoking (OR = 0.031; p = 0.010). The EUR subset showed comparable patterns, with similar effect directions and in some models stronger point estimates despite reduced sample size, consistent with the higher predictive accuracy of PRS in ancestrally matched individuals (Supplementary Table 6). We further assessed PRS distributions by rare variant carrier status across the cohort. Carriers of at least one candidate rare variant in the full cohort (n = 19) had significantly lower PRS than non-carriers (n = 91) (SE = 1.35; OR = 0.060, 95% CI 0.004–0.839; *p* = 0.037; Fig. 3), with stronger associations after adjustment for BMI (OR = 0.0117; *p* = 0.010) and BMI plus smoking (OR = 0.0173; *p* = 0.018). In the EUR subset, effect sizes remained directionally consistent, while most models did not show significance, except for the BMI-adjusted model (OR = 0.0049; *p* = 0.042) (Supplementary Table 6). Across both age-group and carrier-status analyses, models adjusted for fewer ancestry principal components yielded directionally consistent effect estimates, though with attenuated statistical significance.

## Discussion

In this study we investigated the presence of rare variants with potentially large effect sizes, either as candidates for known monogenic IFN-I-IEIs [49] or with potential deleterious effects in GWAS risk genes for COVID-19 severity [14], in a cohort of 110 hospitalized young adults from the NAPKON Study [33]. Consistent with prior findings from the independent German DeCOI study [33], no pathogenic SNVs or large deletions detectable by our CNV analysis were identified in *TLR7*. Other cohorts [18, 20, 21, 23, 54] have reported frequencies of up to ~ 2% likely reflecting differences in cohort composition and ancestry. However, using an extended list of 25 IEI genes involved in type I IFN production and tolerance, we identified seven previously unreported VUS in COVID-19 patients. Since current evidence is insufficient to infer causality, these variants remain of uncertain significance under ACMG criteria and require replication, functional validation and deeper phenotyping to clarify their potential contribution to disease risk.

Among them, a hemizygous missense variant in *IKBKG* in a male individual with severe disease was notable. Although this gene was included in the thirteen extensively studied IFN-I immunity candidate loci for life-threatening COVID-19 pneumonia [17, 18, 21, 31, 54, 55], no variants of potentially damaging impact in this gene have been reported in severely affected COVID-19 patients to date. Pathogenic variants in *IKBKG* are known to cause varying degrees of inactivation of the NF-κB signaling pathway, a key regulator of immune and inflammatory responses, and can lead to a wide range of clinical manifestations. While complete loss-of-function variants in *IKBKG* are lethal in male fetuses and cause dominant incontinentia pigmenti (IP) in females, hypomorphic variants that impair but do not abolish NF-κB signaling are associated with ectodermal dysplasias and immune-deficiency syndromes in hemizygous males [56]. The broad phenotypic heterogeneity of *IKBKG*-related immunodeficiencies [50] makes it an interesting candidate gene in the context of COVID-19 severity.

Furthermore, we identified a heterozygous missense VUS in *AIRE* which warrants functional investigation for a potential dominant-negative effect and involvement in non-classical form of polyendocrine syndrome type 1 (APS-1) that has not been described in COVID-19 patients to our knowledge. Biallelic pathogenic variants in *AIRE*, which is critical for central immune tolerance and the prevention of autoimmunity, cause autosomal recessive polyendocrine syndrome type 1 (APS-1) which has been reported in patients with severe COVID-19 [29–31].However, heterozygous dominant-negative variants, particularly in the PHD1 zinc finger or SAND domains of this protein cause non-classical APS-1 [51, 52] with later onset, milder phenotypes, and reduced penetrance. A recent study similarly hypothesizes a dominant negative effect of missense *TLR7* variants in females with severe COVID-19, highlighting the need for further in vitro functional investigations of these types of variants [23]. This underscores the importance of considering alternative pathomechanisms, inheritance patterns, and variable phenotypes when interpreting rare variants in disease genes.

In line with this, we extended our analysis beyond established inheritance patterns of IFN-I-IEIs and identified 17 additional heterozygous variants in genes currently associated only with recessive inheritance. A similar strategy was applied in the original study of 13 type I IFN related IEIs in severe COVID-19 patients [17], where both monoallelic and biallelic variants in genes were considered and heterozygous possibly deleterious variants in recessive genes (e.g., *UNC93B1, IRF7, IFNAR1, IFNAR2*) were experimentally validated, thereby suggesting novel inheritance patterns. We similarly, detected heterozygous potentially damaging variants in *UNC93B1* (n = 3) and *IRF7* (n = 3), including a frameshift variant (*UNC93B1:* c.699del, p.(Cys234AlafsTer6), gnomAD AF: 0.0001859%) in a 36-year-old male patient with life-threatening disease,and a missense variant (*IRF7:* c.1037C > T, p.(Thr346Met), 0.0003164%) with consistentlydeleterious in silico predictions (CADD: 27.1, Alphamissense: likely_pathogenic, REVEL: 0.771) in a 26-year-old male patient with moderate disease (Supplementary Table 5). We also observed heterozygous pLOF variants in *IFIH1* (n = 3) and *TYK2* (n = 1), monoallelic LOF variants of which have been previously reported in patients with severe COVID-19 [57] and recurrent pulmonary infections [58] respectively. Although reduced penetrance cannot be excluded, the contribution of these variants to autosomal-dominant disease remains uncertain without statistical evidence from case–control analyzes and functional validation. Also, we cannot fully exclude the possibility of undetected compound heterozygous variants in these cases due to technical limitations. And most importantly, although these findings are of interest, based on objective characteristics like allele frequencies, in silico prediction scores or previously established associations with other diseases alone, causality in the context of COVID-19 cannot be assumed.

Genes implicated in COVID-19 severity by GWAS might also harbor rare variants with large effects that could contribute to disease severity in a monogenic fashion, and some of these genes, such as *JAK1*, *OAS1*, *TYK2*, and *IFNAR2* [16], have already been implicated as IEIs. Thus, we analyzed 23 GWAS candidate genes reported in the largest genome sequencing–based study for COVID-19 severity, applying a similar rare variant filtering strategy and identified 13 rare heterozygous variants predicted to be deleterious. Although these findings do not provide evidence for pathogenicity, they might highlight several genes as candidates for future burden testing and functional follow up in larger and independent cohorts. For instance, among the genes with missense variants, *THBS3* showed the highest missense Z-score indicative of partial intolerance to missense variation. We identified two rare missense variants in *THBS3*, including one in the youngest female patient in the cohort with severe disease who required mechanical ventilation upon hospitalization. Although *THBS3* has been linked to COVID-19 susceptibility and severity through GWAS with a potential role in airway mucosal defence during viral entry [14, 16], gene burden analyzes have detected only weak or suggestive rare variant associations, possibly limited by low variant frequency and sample size [21, 54], and no monogenic disease has yet been linked to this gene. A recent spatial proteomics analysis of post-mortem lung tissue from patients with severe COVID-19 showed significant enrichment of THBS3 in fibrotic regions [59], suggesting a potential role of this gene in the development or progression of pulmonary fibrosis. These findings support further investigation into the potential contribution of rare *THBS3* variants to severe COVID-19.

Since both rare and common variants are known to contribute to the complex genetic architecture of COVID-19 severity [60, 61], we took advantage of genome sequencing to perform an exploratory case-only PRS analysis to examine the polygenic burden of disease severity within our cohort. To balance ancestry matching with statistical power, PRS associations were examined in both the full cohort and a PCA-defined European subset, in line with prior evidence showing that reduced ancestry matching predominantly affects predictive performance rather than generating artifactual associations [62, 63]. We observed that younger patients (< 40 years) with severe disease carried higher PRS on average than older individuals, consistent with prior reports that common genetic risk is higher in individuals ≤ 60, both for major risk loci [64, 65] and at the genome-wide level [31]. Moreover, carriers of at least one rare candidate variant in either GWAS or IFN-I-IEI genes tended to show lower PRS than non-carriers. This pattern is in line with recent studies across multiple disorders indicating common-variant background can modulate the effects of rare pathogenic variants, influencing penetrance, expressivity, and clinical outcomes [66–69]. Although our results are limited by small cohort size and cannot support definitive conclusions, the observed patterns suggest an integrated model in which rare variants of large effect and common polygenic background may act together with age and other risk factors to determine COVID-19 severity. Future efforts should focus on jointly analyzing variation across the full allele frequency spectrum and validating findings in larger, well-controlled cohorts using novel statistical methods [60, 70]. NAPKON’s comprehensive dataset, including deep phenotyping, multi-omics, and genetic data available for a subset of participants, will enable future studies to advance our understanding of the genetic and molecular determinants of COVID-19 severity.

Limitations of our study include, first, the lack of functional validation due to which all reported variants remain of uncertain significance, limiting conclusions about their potential contribution to disease risk. Second, given the small sample size, we limited our analysis to genes linked to type I IFN immunity rather than all IEI-associated genes, precluding a comprehensive assessment of IEI as a whole. Third, due to the absence of matched controls, we could not perform PCA-adjusted gene burden testing to assess statistical enrichment of rare variants in cases which represents a major limitation to evaluate the specificity of our findings. Fourth, the CNV analysis was performed only qualitatively and indirectly restricted to the *TLR7* locus, leaving a comprehensive analysis of structural variation unexplored. In addition, although all samples underwent joint variant calling and QC, residual batch effects or coverage variability could still have influenced variant detection, particularly for rare variants in low-coverage exons. In addition, some clinical variables including medication history and SARS-CoV-2 variant information, were not available, limiting evaluation of their potential impact on the results. Finally, while we constructed the PRS from GS-based GWAS data to minimize technical variation, the analysis was conducted in a small, case-only cohort without matched controls, limiting statistical power and precluding estimation of absolute genetic risk. The modest sample size may also increase susceptibility to model instability and potential overfitting; therefore, the findings should be interpreted with caution.

In conclusion, we identified rare candidate variants of possible high impact in IFN-I-IEI genes and GWAS-prioritized genes for severe COVID-19 that represent candidates for future experimental validation and genotype–phenotype studies. Our exploratory PRS analyses suggest that polygenic risk may contribute more substantially to severe COVID-19 in younger individuals, whereas carriers of rare variants tended to show lower polygenic burden. This highlights the need for future integrative genomic approaches in larger well-controlled cohorts to better understand the joint contribution of common and rare variants to severe COVID-19.

## Supplementary Information

Supplementary Material 1.

Supplementary Material 2.

## Data Availability

All data supporting the findings of this study are available within the article and supplementary data files. Participant-level data can be accessed via the NAPKON Use and Access procedure: https://napkon.de/use-and-access.
