# Supplementary material for: Genetic contribution to severe COVID-19 in adults under 60 years without major comorbidities in the German National Pandemic Cohort Network (NAPKON)
Source: Hum Genomics. 2026 Jan 23;20:23. doi: 10.1186/s40246-025-00904-9 (PMC12849158; doi:10.1186/s40246-025-00904-9)
Supplement: Supplementary file 1 — Supplementary Material 1. [file 40246_2025_904_MOESM1_ESM.docx]

**Supplementary Figures:**

**Supplementary Figure 1: Age distribution by disease severity and sex in the cohort.** The distribution of age across different disease severity based on the WHO categories grouped by sex is shown. Hospitalized moderate disease (WHO scores 4–5): hospitalized and no oxygen therapy or oxygen therapy by mask or nasal prongs.; Hospitalized severe disease (WHO scores 6–9): hospitalized and oxygen therapy by noninvasive ventilation, intubation, or mechanical ventilation. The interquartile range (IQR) is represented by each box, with the median age indicated by the horizontal line within the box. Whiskers extend to the minimum and maximum values within 1.5 times the IQR, and outliers are displayed as individual points.

**Supplementary Figure 2. *TLR7* coverage Inspection in 82 male individuals.** Integrative Genomics Viewer (IGV) visualization displaying sequencing read coverage across a ~27 kb region on chromosome X, spanning the *TLR7* gene. The figure consists of two side-by-side panels, each representing data from approximately 40 samples. Each horizontal track corresponds to coverage from a single sample, with gray peaks indicating sequencing depth at each position. The coverage remains consistent and uniform across the entire region, with no significant continuous drops or read absences that would suggest large deletions. The regions highlighted in red correspond to the two coding exons of *TLR7.*

**Supplementary Figure 3. Principal component analysis and ancestry inference.** Principal component analysis of NAPKON genomes merged with the 1000G reference panel. Only the first two principal components are shown, with the 1000G reference individuals (grey) shaped by super-population labels used for random forest training. Study samples are overlaid and colored according to ancestry assignments obtained from the RF classifier, which uses the first 20 PCs as input features and assigns the class with the highest vote. AFR: African, AMR: Admixed American, EAS: East Asian, EUR: European, SAS: South Asian.
